# Lectures on Military Surgery

**Published:** 1861-09

**Authors:** E. Andrews

**Affiliations:** Professor of Surgery, Surgeon to the Mercy Hospital; to the Chicago Med. Dispensary, etc.


### CHICAGO MEDICAL EXAMINER

N. S. DAVIS, M. D., Editor.---FRANK W. REILLY, M. D., Ass't Editor.

VOL. II.] SEPTEMBER, 1861. [NO. 9.

### Original Contributions.

ARTICLE I.

### LECTURES ON MILITARY SURGERY:

DELIVERED DURING THE SUMMER COURSE OF THE MEDICAL DEPARTMENT OF LIND UNIVERSITY.

By E. ANDREWS, A. M., M. D.,

PROF. OF SURGERY; SURGEON TO THE MERCY HOSPITAL; TO THE CHICAGO MED. DISPENSARY, ETC.

### LECTURE FIFTH.

TREATMENT OF GUN-SHOT WOUNDS.

*Extraction of the Bullet.*—The skin opposes more resistance to the passage of a ball than any other soft tissue, hence, in a large proportion of the wounded, the projectile, after having traversed the whole limb or body, is found just beneath the skin, on the side opposite the entrance. In all such cases the best way is to make a small incision, and extract the offender. If, however, the bullet is not thus favorably located it will become necessary to employ other measures for its removal. For this purpose a variety of instruments have been invented. The most common is the bullet-forceps. This is a pair of forceps from eight to fourteen inches in length, perfectly straight, and small enough to follow in the track of the projectile. The jaws should be slender and at least four inches in length, and terminate either in teeth or cup-shaped cavities, to hold the ball. The instrument is introduced tightly closed, until the end strikes the bullet, when the blades should be separated a little, slipped over it, and withdrawn. The proper seizure, however, is often difficult. The bullet may be imbedded in spongy bone, or what is still more embarrasing, it may be lodged behind folds of fasciæ in such a way that it can only be seized with them, and therefore cannot be drawn out. In such cases, patience and perseverance will generally succeed after a time. An objection to the ordinary forceps is, that it requires to be pushed too far on either side of the ball before it will hold, and then the teeth are liable to embrace the adjoining tissues in their grasp. To evade this difficulty, several devices have been resorted to. Tiemann & Co. have constructed a pair in which the blades are slender, and terminate each in a single round tooth, sloping forward, with no cup. The advantage of this is, that the teeth, when slightly separated, may be sunk firmly into the lead on the posterior side of the ball, and do not have to be pushed past it so as to grasp its bulge. Another instrument consists of a simple rod, terminating in a screw-point like a gimlet, which being turned into the lead a short distance, will hold fast and readily bring it away. In circumstances where apparatus must be extemporized, a common gimlet, or anything having a similar point, will often be an available extractor. In some instances, folds of cloth are driven before the ball in such a way, that by drawing them carefully out, the projectile will come with them. If a bullet cannot be reached or extracted without very extensive or dangerous incisions, it should be allowed to remain. Frequently, by the progress of suppuration and granulation, it will, after a time, be brought within reach, and if not, it often remains without injury or irritation, imbedded in the flesh. I have extracted a nnmber of old shots from the flesh of subjects in the dissecting-room, without being able to discover any evidence of inflammation in the adjacent tissues. Other foreign bodies must be removed still more carefully then bullets, because they offer less hope of remaining innocuous in their beds. Pieces of bone, shreds of clothing, fragments of shell, splinters and stones are among the objects requiring attention.

*Hæmorrhage.*—In gunshot wounds, the primary hæmorrhage is usually slight. The exceptions to this rule occur when large vessels happen to be cut in the passage of the ball. In such cases the flow of blood may be very large or even immediately fatal. Simple field tourniquets, consisting of a strap, buckle and pad, should be provided in numbers sufficient for the purpose, besides those used in operations. If none are at hand at the moment, one may be extemporized by taking a cord, strap, or sash, tying it about the limb with a wad of clothing placed on the artery for a compress, and then twisting the ligature tight by means of a stick. In the Crimea, attempts were made to acquaint common soldiers with the tourniquet, so that they might, in emergency, save life by applying them to each other. The popularity of this humane project received a shock by the discovery that one soldier had applied a tourniquet *around the neck* of a comrade who was bleeding freely in that region. Fortunately he was alarmed at the symptoms of suffocation, and loosened the strap before death resulted. The persulphate, or the perchloride of iron may be applied with good effect to the bleeding vessels in some instances. While primary hæmorrhage is less frequent in gun-shot than in incised wounds, the secondary hæmorrhage is more common. This occurs generally between the fifth and fifteenth days, and during that time, not only should proper watchfulness be observed, but great care should be taken that the patient is not moved about, nor allowed to exert himself unnecessarily. In the Crimea, the simple removal of the patients from the camp before Sebastopol to Balaklava, produced a number of fatal cases. If secondary hæmorrhage occurs copiously, it may, in some instances, be checked by astringents and compression, but very often it will be found necessary to tie the artery above the wound.

*Gun-shot Wounds in the Head.*—Are usually fatal if they penetrate the brain. It is true that some astounding recoveries have been made, as for instance, in the well-known case where a tamping-iron was blown through the skull of a man, without killing him. Such cases serve to caution us not to relax our efforts, nor abandon, too soon, all hope of a patient; but, nevertheless, such cures are very rare, and the usual rule is, that a bullet penetrating the brain, causes death. In wounds of this organ, it is generally not possible to extract the ball. Still a very cautious probing should be made, bearing in mind the softness of the brain-substance, and if the bullet can be found, it should be removed. To accomplish this, it will be required to enlarge the opening in the skull with the trephine, or gouge-forceps, as the orifice will not admit of the ball and the additional thickness of the forceps being withdrawn through it. After removing all foreign bodies which are safely accessible, the patient must be treated as for any other case of compound fracture of the skull. If he survives the first shock of the injury, he will have to confront the dangers of inflammation of the brain, and of *hernia cerebri*, and will need venesection, cold applications, etc., as in civil surgery.

*Gun-shot Wounds of the Chest.*—These are often fatal from a wound of the heart, or of the large vessels. It is not true, however, that every wound of the heart produces immediate, or even ultimate death. The case of the notorious Bill Poole, of New York, is an instance where the patient survived many days with a pistol wound in the heart. Many other instances have occurred equally striking, and in some of them when death had taken place years afterwards, from other causes, a post mortem examination has shown the heart perfectly healed and presenting a distinct cicatrix. Such instances occur only when the organ has been slightly touched by a small shot which has not seriously disorganized it. The almost universal rule is, that a gun-shot wound in the heart is immediately fatal. The treatment of the slight cases which prove otherwise, is repose, atiphlogistic, and in some instances, cardiac sedatives, such as veratrum viride.

Less fatal, but still fearfully dangerous are the wounds which penetrate the lungs. The bullet in these cases entering into the pleural cavity, or at least, the soft, unresisting tissue of the lung, is seldom stopped near the orifice, but either passes quite through the body, or lodges near the opposite side. No deep and perilous exploration, therefore, should be made for it. As a general rule, these cases where the bullet passes quite through the body, are safer than those where it lodges in the lungs. Where both lungs are exposed by the wound, they at once collapse and asphyxiate the patient; but where one cavity alone is exposed, the collapse does not take place, except in certain cases. On the contrary, it is not rare to see the lung bulge, like a hernia, from a large wound at each expiration. This phenomenon wherein wounds of the living body differ from those of the dead, has been explained in various absurd ways. The truth appears to be this: The sound lung continues to draw in air without impediment, but in expelling it, it is driven out with a slight pressure, especially if the patient narrows the glottis by sighing and groaning. The pressure thus induced, forces the air into the lung on the wounded side, and expands it at each expiration, which it only partially contracts at each inspiration, thus keeping up the contact between the viscus and the pleura. If the patient bleeds very freely, it will be expedient to lay him with the wounded side down, if he can tolerate the position, in order to allow the blood to drain out of the chest. If there is evidence that the lung is wounded, it will not be safe to close the wound, even after the hæmorrhage has ceased, as in such circumstances the air sometimes accumulates in the pleura to such an extent as to greatly distress the patient. The mechanism of the accident seems to be this: The wound in the lung may be valvular in form, in which case every expansion of the chest sucks air freely into the pleura from the bronchi, while the contractions, owing to the valve shape of the wound, are not able to expel it. If, now, the external wound be tightly closed, there is no place of escape left, and the air pumped into the pleura will accumulate at each inspiration, until its pressure threatens asphyxia. In a very few cases there may be troublesome hæmorrhage from an intercostal artery. If it does not cease upon pressure, it may be easily checked by ligation, either at the wound, or by cutting down posterior to it, and carrying a curved needle under the vessel. Some have recommended passing a ligature around the rib, so as to compress the artery against it, but this is unnecessary and dangerous to the pleura. The subsequent treatment consists in abating the inflammation of the lungs and pleura, by appropriate measures, bearing in mind that the patient will probably have to endure a long and exhaustive suppuration, and hence, he should not be bled, unless the necessity is very urgent. In the suppurative stage, the strength must be supported by generous diet and tonics, and in a portion of the cases, you will have the satisfaction of carrying your patient through to a complete recovery.

*Wounds of the Abdomen.*—These are quite as dangerous as those of the chest. In many cases the hæmorrhage from the large vessels is the cause of death, while in others the perforation of the hollow viscera and the subsequent peritonitis produce more slowly the same result. The bullet generally, cannot be found. It does not necessarily follow that a ball entering the abdominal walls, has penetrated the cavity. It may glide around in the interfascial or in one of the intermuscular spaces, and do but little injury. If it enters the cavity, there is still a bare posibility that it may have dropped among the viscera without wounding them. Shot have been known to penetrate the intestines, and be discharged with the fæces without fatal results, and the well-known case of Alexis St. Martin shows that a pistol shot may lay open the stomach without death. In spite of exceptional cases, however, most of these wounds are fatal. There is comparatively little that the surgeon can do for the wounded man, except in a few rare cases. I can only say therefore, that the treatment is the same as is laid down for your guidance in works on civil surgery. In a very few cases you may be able to save life by making an artificial anus.

*Wounds of the Pelvis.*—Gun-shot wounds fracturing the pelvis, and wounding severely its viscera, are always, or nearly always fatal. A few cases of wounded bladder have recovered. If the urinary organs are injured, it will be requisite to keep a catheter in the bladder, and if urinary infiltrations occur, free incisions must be made to evacuate the fluid.

*Bayonet stabs* of the trunk are less dangerous than bullet wounds. The viscera, particularly the intestines, often glide away from the blunt point of the weapon, and allow it to pass without injury. They are to be treated on general principles.

*Gun-shot wounds of the Extremities.*—These afford the best field for the exercise of skill and judgment by the surgeon.

If the wound be simple, not involving important bones, nerves or vessels, it may be treated with cold-water dressings. If it is more serious in its nature, the question of amputation presents itself for immediate decision. It is necessary that the army surgeon be prepared to make up his mind on this point with promptness. I shall therefore endeavor to condense the principles involved, into a simple and easily remembered form:

1. Owing to the fact that the wounded must often be transported in wagons, and in various other respects, can be less perfectly cared for, than in civil life, some limbs must be amputated, which a private practitioner would save.

2. It will be justifiable to go further in conservative efforts to avoid amputation in the superior, than in the inferior extremity, because the limb being smaller, suppuration and gangrene are less fatal, and the final results of a secondary amputation are less to be dreaded.

3. Simple flesh wounds, simple fractures, and moderately severe compound fractures do not require amputation.

4. If a moderate compound fracture be accompanied with a laceration of the artery, while the vein and nerve are safe, amputation is not required.

5. Compound fractures of large limbs with extensive destruction of soft parts, especially of the nerves or vessels, usually require amputation. Hamilton says, and others agree with him, that compound fracture of the femur, even if the nerves and vessels are safe, requires amputation; but the mortality after amputation of the thigh is so fearful in military surgery, that I advise some discretion in applying the precept.

6 Gun-shot wounds of the knee and ankle joint require amputation. Those of the elbow, wrist and shoulder, require amputation or resection. Those of the tarsus generally require amputation.

7. As all military amputations at the hip-joint have been fatal, hitherto it will be best in gun-shot fractures of the superior extremity of the femur, to prefer excision of the head, which is a far less severe operation.

8. Wounds of the fingers and toes may be treated as in civil surgery.

*The Time of Amputation.*—There is a period of a few hours immediately after the injury when the patient is under the influence of the "shock." If the shock be severe, no effort should be made to amputate until reaction occurs.

After the shock is over, the question arises, whether primary or secondary amputation is to be preferred. Primary amputation is that which is performed during the first stages of the injury, that is, within the first days. Secondary amputation is that which is delayed until the period of suppuration sets in.

After considerable discussion, the accumulation of experience gives the following precepts for your guidance:

1. In proportion as your hospital approaches the condition of civil practice, just in that proportion you will be able to give preference to secondary amputation.

2. In proportion as the hospital is overcrowded, and illy ventilated, early amputation will be more necessary.

3. In injuries of the upper extremity, primary amputations are to be preferred; but in those of the lower extremity, the preference is less strong. The results of experience are that in military surgery, primary, and in civil surgery, secondary amputations are to be preferred for the lower extremities.

4. In certain cases, as where the limb is quite destroyed by a cannon ball, primary amputations are to be preferred, even in the upper extremities.

5. In small limbs, as fingers and toes, primary amputation is objectionable, if delay furnishes any hope of saving the part, because the accidents of suppuration and gangrene in these cases are not seriously dangerous, and therefore may be braved.

*The Place of Amputation.*—The rule in military, as in civil surgery is, to amputate as far from the body as the condition of the limb and the facilities for forming a good stump will permit. It is found that the nearer the body the operation is made, the greater is the mortality. Thus in the Crimean war, the mortality after the amputations of the smaller toes was four per cent.; after the large toe, sixteen per cent.; after amputation of part of the foot, twenty-five per cent.; after amputation of the leg, fifty-five per cent.; amputation at the lower third of the thigh, fifty-six per cent.; at the middle third, sixty per cent.; at the upper third, eighty-six per cent.; at the hip joint, one hundred per cent.

All cases of amputation of the hip joint, died without exception. On this account, I advise that no amputation be performed at that joint, if there is the least hope of life by any other treatment. If the head of the femur is shattered, it will be much safer to excise it, which is an operation of comparatively little danger; but the limb should not be cut off if there is the least chance of its preserving its vitality.

In other respects the principles for selection of location are the same as in civil practice.

*Mode of Operation.*—If great haste is required, the flap operation should be preferred, but if time is afforded, and if the patients must be transported far in wagons, the heavy flaps of that operation are objectionable. They shake, jar and tear out the sutures and adhesions by the jolting, and hence the lighter flaps of the circular method are more successful.

*Dressings of Gun-shot Wounds.*—The wounds being lacerated and contused, generally inflame and suppurate somewhat extensively, which fact led former observers to attribute a poisonous effect to the gunpowder. If amputation is not required, the wound after cleansing, is best treated by cold water dressings. These may be applied as ice bags, as wet cloth, or by irrigation. This mode of treatment will be found extensively available and of immense value during the first few days after the injury. After that, simple cleansing and dressings with cerate or lint are required. It is of the utmost consequence that *careful and frequent cleansing* be attended to, as the decomposing discharges of great numbers of wounded in a hospital emit poisonous exhalations, which give origin to hospital gangrene and erysipelas. In all other respects the care of these wounds is as in ordinary surgery. In this matter, the welfare of your patients will depend not so much upon brilliant skill in using the knife, as upon your watchfulness and industry, and determination in enforcing cleanliness.

